# Physical Fitness in Ambulatory Patients with Chronic Lymphoid Malignancies Receiving Monoclonal Antibody-Based Therapy: A Case–Control Study

**DOI:** 10.3390/cancers18132040

**Published:** 2026-06-24

**Authors:** Małgorzata Pudełek, Jarosław Dybko, Iwona Malicka

**Affiliations:** 1 Lower Silesian Oncology, Pulmonology and Hematology Center, 53-439 Wroclaw, Poland; jaroslaw.dybko@dcopih.pl; 2 Department of Physiotherapy, Wroclaw University of Health and Sport Sciences, 51-612 Wroclaw, Poland; iwona.malicka@awf.wroc.pl

**Keywords:** hematologic malignancies, physical fitness, outpatient treatment, rehabilitation

## Abstract

**Introduction:** Hematologic malignancies account for a significant proportion of the global cancer burden. Immunotherapy is currently one of the key treatment modalities used in the management of these diseases. **Objective**: This study aimed to assess the physical fitness of patients with chronic lymphoid malignancies receiving monoclonal antibody-based therapy compared with healthy individuals. **Materials and Methods**: The study included 99 ambulatory patients being treated for hematologic malignancies—33 with chronic lymphocytic leukemia, 32 with multiple myeloma, and 34 with follicular lymphoma—as well as 43 healthy individuals. All participants underwent the Two-Minute Step Test, the 30-Second Sit-to-Stand Test, and the Timed Up and Go Test. **Results**: Patients with hematologic malignancies, regardless of diagnosis, were characterized by significantly lower lower-limb strength (H(3, N = 142) = 24.779, *p* < 0.0001), as well as poorer agility and dynamic balance (H(3, N = 142) = 24.993, *p* < 0.0001). Patients diagnosed with multiple myeloma and follicular lymphoma also exhibited lower cardiorespiratory endurance (H(3, N = 142) = 13.223, *p* = 0.0042). Age was a significant predictor of physical fitness. However, in an analysis treating diagnosis as a categorical variable with the control group as the reference category, patients with hematologic malignancies also had significantly lower physical fitness scores than controls. **Conclusions**: Patients with chronic lymphoid malignancies receiving monoclonal antibody-based therapy exhibit reduced physical fitness regardless of hematologic diagnosis. Patient age is an additional factor associated with physical fitness.

## Simple Summary

Given the increasing incidence of hematologic malignancies worldwide and the growing number of long-term survivors, maintaining functional capacity has become an important clinical concern. The patients included in the present study represent a population that may be particularly vulnerable to treatment-related declines in physical functioning. They demonstrated reduced lower-body strength, poorer balance and agility, and, in some cases, lower exercise capacity. Older age was also associated with worse physical performance. These findings suggest that patients with hematologic malignancies may require additional physical support during treatment. Impairments in physical fitness may negatively affect patients’ independence, quality of life, and ability to tolerate treatment. Therefore, regular assessment of physical performance and psychophysical status should be considered an integral part of comprehensive cancer care. Early identification of functional limitations may facilitate the implementation of individualized rehabilitation and supportive interventions aimed at improving treatment outcomes and overall well-being.

## 1. Introduction

Hematologic malignancies account for a significant proportion of the global cancer burden, representing approximately 6.6% of cases. Each year, more than 1.2–1.3 million new cases of these diseases are diagnosed worldwide. The most common hematologic malignancies include lymphomas, myelomas, and leukemias. These diseases also generate significant economic costs, including direct expenses related to diagnosis and treatment and indirect costs from lost work and long-term patient care [1,2].

Immunotherapy is currently one of the primary treatments for hematologic cancers. Its introduction has led to substantial improvements in treatment outcomes and patient survival [3]. Several immunotherapeutic approaches are used in clinical practice; in hematologic oncology, monoclonal antibody therapy is commonly administered as monotherapy or combination immunochemotherapy. Although targeted therapies are highly effective, they can still be associated with adverse effects. The most common adverse effects reported include anemia, fatigue, difficulty concentrating, memory impairment, bone and joint pain, muscle cramps, anxiety, and depression [4,5,6]. Ultimately, these symptoms can adversely affect both physical fitness and quality of life.

Recently, there has been growing interest in assessing physical fitness in patients with hematologic malignancies. The importance of this assessment is multifaceted. First, it can inform the selection of an appropriate treatment intensity. Patients in good physical condition can utilize more intensive treatment regimens, potentially increasing the chances of remission but also increasing the risk of complications. Conversely, in patients with reduced physical fitness or in older patients, less intensive therapies may be necessary, with a greater emphasis on disease control and quality of life rather than cure [7,8]. Second, physical fitness assessment enables the identification of individuals at risk of complications, allowing for the earlier implementation of preventive measures [9,10]. Third, it serves as a starting point for planning rehabilitation and supportive interventions, such as physical therapy or physical activity programs [11].

Furthermore, measures of physical fitness correlate with survival time [12]. In patients undergoing treatment for malignant tumors, physical fitness levels are an important predictor of mortality [13]. A review of the literature indicates that physical fitness is most commonly assessed in patients undergoing hematopoietic cell transplantation [10,14,15,16]. In contrast, patients treated on an outpatient basis are less likely to undergo such diagnostic testing, and the lack of systematic assessment of their physical functioning results in limited access to rehabilitation programs designed for this patient population.

Therefore, this study aimed to assess the physical fitness of patients with chronic lymphoid malignancies receiving monoclonal antibody-based therapy compared with healthy individuals.

## 2. Materials and Methods

### 2.1. Participants and Recruitment

The study included 99 patients recruited from the Hematology and Transplant Centre at the Lower Silesian Centre for Oncology, Pulmonology, and Hematology in Wrocław—33 diagnosed with chronic lymphocytic leukemia, 32 diagnosed with multiple myeloma, and 34 diagnosed with follicular lymphoma—as well as 43 healthy individuals who had never been treated for cancer and had no comorbidities. The mean age of all participants was 59.71 ± 14.52 years, the mean height was 168.53 ± 8.96 cm, and the mean weight was 77.93 ± 15.97 kg. The mean BMI was 27.34 ± 4.76.

The inclusion criteria for the study group were: age > 18 years, diagnosis of hematologic malignancy, eligibility for outpatient treatment with monoclonal antibodies as monotherapy or combination immunochemotherapy, and written informed consent to participate in the study.

The inclusion criteria for the control group were: age > 18 years, good health, and no history of malignant disease or other chronic conditions. The absence of chronic conditions was determined based on participants’ self-reported medical history obtained during the screening process.

The exclusion criteria included intellectual disability and significant impairments in motor function (e.g., gait, balance, or mobility disorders) that could potentially affect the assessed parameters or refusal to participate in the study.

A consecutive sampling strategy was employed. Recruitment was conducted between January 2024 and May 2025. Eligible participants were enrolled in the order in which they were identified during the recruitment period, provided they met the inclusion criteria and consented to participate in the study.

### 2.2. Research Methods

Tests with high predictive value were used to assess physical fitness and to plan, monitor, and evaluate the effectiveness of physical activity interventions [9,17,18,19]. Cardiorespiratory endurance was assessed using the 2MST; lower-limb strength, using the 30-Second Sit-to-Stand Test; and agility and dynamic balance, using the Timed Up and Go Test.

• The Two-Minute Step Test (2MST) assesses exercise tolerance. On the signal “start,” the subject begins walking in place with their right leg, lifting each leg alternately to a height corresponding to the midpoint of the thigh. The subject may use a wall, chair, or table for support to maintain balance. The outcome measure was the number of times the right knee was raised.
• The 30-Second Chair Stand Test (30CST) assesses lower-limb strength, which is required for many activities, including climbing stairs, walking on uneven ground, standing up from a chair, and getting in and out of a car. The test involves performing as many sit-to-stand repetitions as possible within 30 s with the arms crossed over the chest. The outcome measure was the number of repetitions completed within 30 s.
• The Timed Up and Go Test (TUG) assesses agility and dynamic balance, which are important in activities requiring quick maneuvers or rapid motor decision-making, such as getting off a bus or walking to the bathroom or kitchen. The test involves rising from a seated position, walking 3 m, turning around, walking back, and returning to a seated position. The outcome measure was the total time required to complete the task correctly without loss of balance.

Sociodemographic information was obtained using a questionnaire that assessed participants’ educational background, place of residence, marital status, employment status, and other basic personal characteristics. In addition, clinical data were collected for patients with hematological malignancies, and fatigue severity was assessed using the Fatigue Severity Scale (FSS). The Fatigue Severity Scale (FSS) is a unidimensional instrument comprising nine items rated on a seven-point Likert scale, where higher scores reflect greater fatigue severity. The scale has shown satisfactory test–retest reliability and high internal consistency across diverse clinical populations.

### 2.3. Ethics

This study was approved by the Senate Research Ethics Committee, Wrocław University of Health and Sport Sciences. Consent number: 28/2023; date of approval: 12 January 2024.

### 2.4. Statistical Analysis

The distribution of the data was verified using the Shapiro–Wilk test. Due to the non-normal distribution of the analyzed variables, the median was used as a measure of central tendency, and the interquartile range (IQR) as a measure of variability. Differences between groups were assessed using the Kruskal–Wallis test followed by Dunn’s post hoc test with Bonferroni–Holm correction.

To assess the effect of diagnosis on physical fitness, the results of the three fitness tests were standardized using z-scores based on the mean and standard deviation calculated for the entire study sample. For the Timed Up and Go test, in which shorter completion times indicate better performance, the direction of the scale was reversed prior to standardization. Subsequently, the arithmetic mean of the three standardized scores was calculated to derive an overall functional performance index. Higher values of the index indicated better functional performance.

A linear regression model was then applied, with diagnosis included as a factor and age, sex, and body mass index (BMI) included as covariates. The significance level was set at *p* < 0.05. Effect sizes were determined using eta squared (η^2^) and interpreted as follows: small effect, η^2^ ≈ 0.010; medium effect, η^2^ ≈ 0.060; and large effect, η^2^ ≈ 0.140 [20].

An additional analysis was conducted in the subgroup of patients with hematological malignancies, following the exclusion of healthy participants who had previously constituted the control group. The standardized physical functioning score was used as the dependent variable. Diagnosis was entered as a categorical predictor, with Leukemia serving as the reference category.

The extended multivariable model included diagnosis, age, sex, body mass index (BMI), educational attainment, log_2_-transformed treatment duration, Fatigue Severity Scale (FSS) score, and the number of comorbidities as explanatory variables. Treatment duration was log-transformed prior to analysis because of its markedly right-skewed distribution. Model parameters were estimated using ordinary least squares (OLS) linear regression with HC3 heteroscedasticity-consistent standard errors.

Statistical analyses were performed using Statistica version 13.1 (StatSoft, Inc., Tulsa, OK, USA) and an online tool for calculating effect sizes available at Psychometrica (https://www.psychometrica.de/effect_size.html; accessed 26 January 2026).

The sample size was estimated using G*Power version 3.1.9.2 (Heinrich-Heine University Düsseldorf, Düsseldorf, Germany). The composite functional performance index was defined as the primary study endpoint. The primary analysis was performed using a multivariable linear regression model. In addition, the Kruskal–Wallis test was used as a supplementary method to assess unadjusted differences between the groups. Assuming a moderate effect size (*f*^2^ = 0.15), a significance level of α = 0.05, and a power of 0.95, the required total sample size was 129 individuals. The sample size was increased to account for potential missing data, assuming a 10% defect rate, and 142 participants were ultimately recruited.

## 3. Results

No significant differences in age or body mass index (BMI) were observed among the study participants. However, significant differences were found in education level and employment status. Detailed results for the analyzed variables are presented in Table 1.

Except for disease-specific treatment regimens, no significant differences were observed in the clinical characteristics of the identified patient groups. Detailed results for the analyzed variables are presented in Table 2.

Patients with hematologic malignancies, regardless of diagnosis, exhibited significantly lower lower-limb strength (H(3, N = 142) = 24.779, *p* < 0.0001) and poorer agility and dynamic balance (H(3, N = 142) = 24.993, *p* < 0.0001) compared with the control group. Detailed results are presented in Table 3 and Table 4.

Patients diagnosed with multiple myeloma and follicular lymphoma also exhibited lower cardiorespiratory endurance compared with the control group, H(3, N = 142) = 13.223, *p* = 0.0042. Detailed results are presented in Table 3 and Table 4.

The linear regression model was statistically significant (*F* = 18.53, *p* < 0.001) and explained 45.2% of the variance in physical fitness (R^2^ = 0.452).

Age was a significant predictor of physical fitness (β = −0.028, 95% CI [−0.035, −0.020], *p* < 0.001), with a very large effect size (partial η^2^ = 0.27).

In an analysis treating diagnosis as a categorical variable, with the control group as the reference category, patients with leukemia, (β = −0.49; 95% CI [−0.80, −0.17], *p* = 0.003), myeloma, β = −0.79; 95% CI [−1.11, −0.48], *p* < 0.001, and lymphoma, β = −0.57; 95% CI [−0.88, −0.27], *p* < 0.001) achieved significantly lower physical fitness scores than the control group. The effect sizes for these variables ranged from small to moderate, partial η^2^ = 0.06–0.16.

Sex, β = 0.20; 95% CI [−0.02, 0.42], *p* = 0.078, and BMI, β = −0.022; 95% CI [−0.046, 0.002], *p* = 0.067, were not statistically significant predictors of physical fitness; however, non-significant trends toward higher fitness in women and lower fitness with increasing BMI were observed.

Predictors of physical fitness are presented in Table 5.

In the patient cohort, an additional multivariable linear regression analysis was performed, incorporating diagnosis, age, sex, BMI, educational level, logarithmically transformed treatment duration, FSS score, and the presence of comorbidities as explanatory variables. The extended model was statistically significant overall (F = 6.33, *p* < 0.001) and explained approximately 39.3% of the variance in functional status (R^2^ = 0.393; adjusted R^2^ = 0.331). After adjustment for the additional covariates, age emerged as the only significant independent predictor of functional status (β = −0.0277, *p* < 0.001).

The comparison of Myeloma vs. Leukemia showed a negative association with functional status; however, this effect did not reach statistical significance (β = −0.3183, *p* = 0.091). None of the remaining variables included in the extended model—sex, BMI, educational attainment, log_2_-transformed treatment duration, FSS score, and the presence of comorbidities—were significant independent predictors of functional status in the multivariable analysis (Table 6).

## 4. Discussion

It has been projected that the number of individuals diagnosed with and dying from cancer, including hematologic malignancies, will continue to rise. In this context, it is essential to provide this patient population with comprehensive care, including appropriate exercise programs as an adjunctive treatment throughout the course of care [21]. The development of exercise programs requires prior assessment of physical fitness.

Physical fitness is an important indicator of the functioning of the cardiovascular, musculoskeletal, and nervous systems. It reflects the efficiency of oxygen transport and utilization and affects mental well-being and quality of life [22].

Monitoring the physical fitness of patients with hematologic malignancies receiving outpatient treatment may facilitate assessment of their ability to perform activities of daily living and to live safely and independently at home [21]. Reduced physical fitness may increase the risk of falls, injuries, or excessive fatigue, which can be particularly dangerous in the home environment [23].

In the present study, patients with hematologic malignancies demonstrated significantly lower levels of physical fitness than healthy individuals. A significant difference was observed in the 30CST and the Timed Up and Go Test, regardless of diagnosis, and in the 2MST among patients with myeloma and lymphoma. Differences in physical fitness levels between the control group and groups diagnosed with hematologic malignancies—leukemia, myeloma, and lymphoma—were also confirmed by a linear regression model. Deficits in physical fitness may translate into limitations in performing activities of daily living, an increased risk of falls, and a decline in overall functional capacity. Notably, the 30-s Chair Stand Test scores observed in our cohort (9–11 repetitions) were at the lower end of published age-adjusted normative ranges for older adults, suggesting reduced lower-extremity strength and functional performance. Similarly, the Two-Minute Step Test results (82.5–89 knee raises) were below average values reported for healthy adults of comparable age, indicating diminished functional endurance and aerobic capacity. Although the mean Timed Up and Go values (8.10–9.45 s) remained below commonly accepted thresholds associated with an increased risk of falls, the poorer performance observed among patients with hematologic malignancies may nevertheless reflect reduced mobility. Collectively, these findings suggest that the observed differences are not only statistically significant but also clinically relevant, reflecting impairments in physical function that may adversely affect independence, activities of daily living, and quality of life. They may also indirectly reflect the degree of sarcopenia-associated muscle wasting [24]. In a previous analysis of the same patient cohort, we found that, irrespective of age, patients exhibited lower levels of physical activity and accumulated more sedentary time than healthy controls. These findings complement the present results by providing behavioral context for the objectively assessed impairments in functional fitness observed in this population [25].

Particular attention should also be paid to patient age, which has been shown to be a significant independent predictor of physical fitness. A gradual decline in overall physical fitness is observed with increasing age. Furthermore, age may exacerbate the processes leading to the development of sarcopenia, defined as the progressive loss of skeletal muscle mass and function. This phenomenon, combined with the burden of the disease and its treatment, may further limit patients’ independence, increase the risk of falls, and impair their overall quality of life [26,27].

One of the most common and debilitating problems in cancer treatment is fatigue, which affects 60–100% of patients during or after treatment and is particularly prevalent among patients with lymphoma and leukemia. To minimize fatigue, patients usually avoid physical exertion. Vermaete et al. and Xu et al. suggested that lower physical fitness in this patient group may be associated with greater fatigue [28,29]. However, no such association was observed in our study.

Multiple myeloma is characteristically associated with bone disease; bone involvement is observed in approximately 79% of patients with newly diagnosed myeloma. This can affect physical function [30].

The linear regression model demonstrated a significant association between a diagnosis of hematologic malignancy and physical fitness. Assessing physical fitness enables the identification of patients at risk and the implementation of interventions to prevent a decline in physical functioning or to improve physical fitness. Maintaining or improving physical fitness is essential for enabling patients to perform activities of daily living and maintain a high quality of life [30].

Consequently, regardless of diagnosis or outpatient treatment regimen, all patients should undergo a functional assessment, the results of which should serve as a basis for rehabilitation. Consistent with American College of Sports Medicine (ACSM) and the American Cancer Society (ACS) guidelines, patients should be encouraged to participate in 150–300 min of moderate-intensity, low-impact aerobic activity per week (e.g., walking or cycling), complemented by resistance training twice weekly, with a particular focus on lower-limb muscle strengthening to help maintain physical function. Regular exercise programs for patients with cancer may support independence in daily functioning, including the ability to perform activities of daily living and participate in work. Multidisciplinary interventions, including physical components, have been shown to increase return-to-work rates [31].

Given the significant differences observed between patients with hematologic malignancies and healthy controls in terms of education and employment, these factors may also warrant consideration when interpreting physical fitness and functional outcomes. In the case of employment, one possible explanation is that patients with hematological malignancies may retire earlier because of disease-related limitations or eligibility for retirement and disability benefits. Consequently, employment patterns in the patient group may differ from those observed in the healthy control group.

## 5. Limitations

The main limitations of the study include the small sample size and the heterogeneity of the study group, resulting from the variety of diagnoses among patients with hematologic malignancies, which may limit the generalizability of the findings.

Parameters related to sarcopenia, such as skeletal muscle mass and handgrip strength, were not assessed in the present study. These factors may have influenced the functional performance of the participants. In addition, laboratory parameters, particularly hemoglobin concentration, inflammatory markers, and indicators of nutritional status, are known to significantly affect exercise capacity and muscle function. The inclusion of these measures in future studies would provide a more comprehensive understanding of the determinants of physical status.

## 6. Future Research Directions

Future studies should include larger, more homogeneous patient groups stratified by specific hematologic malignancies. This would allow a more precise assessment of differences in physical fitness levels and functional limitations characteristic of individual conditions.

It is also important to extend the research to include assessment of sarcopenia, encompassing measurements of muscle mass and strength as well as physical fitness. This would provide a better understanding of the mechanisms underlying functional limitations and their relationship to the disease course and treatment.

Another important consideration is the inclusion of biomarkers such as hemoglobin levels, given their potential impact on oxygen delivery, exercise tolerance, and physical performance in patients with hematologic malignancies.

At the same time, it should be noted that the assessment of physical fitness in this study was conducted in an outpatient setting, which remains relatively uncommon and is not well described in the literature. To date, most studies have focused on hospitalized patients or those in strictly controlled clinical settings. In contrast, assessments conducted in an outpatient setting may better reflect patients with hematologic malignancies’ actual functioning in everyday life. It is therefore appropriate to conduct further research on the standardization of methods for assessing physical fitness in outpatient care, including their prognostic value and clinical utility in patients with hematologic malignancies.

## 7. Conclusions

Patients with chronic lymphoid malignancies receiving monoclonal antibody-based therapy exhibit reduced levels of physical fitness, regardless of the type of hematologic malignancy diagnosed. Another factor associated with physical fitness is patient age. Consequently, rehabilitation programs should be considered for this patient population, with particular emphasis on interventions designed to improve lower-limb strength, agility, and dynamic balance, tailored to both the specific condition and the patient’s age.

## Figures and Tables

**Table 1:** Sociodemographic characteristics of the study groups.

Variable	Leukemia	Myeloma	Lymphoma	Control Group	*p*-Value
***n***	33	32	34	43	
**Age,** years; median (IQR)	61.00 (22.00)	66.50 (11.50)	64.50 (20.00)	58.00 (26.00)	0.06 ^a^
**BMI**, kg/m^2^; median (IQR)	25.83 (4.6)	25.89 (6.07)	26.76 (6.17)	27.53 (7.58)	0.10 ^a^
**Sex, %**					0.15 ^b^
Female	42.42	56.25	44.12	65.12	
Male	57.58	43.75	55.88	34.88	
**Education, %**					0.03 *^b^
Primary	6.06	9.68	11.76	4.65	
Vocational	27.27	22.58	23.53	11.63	
Secondary	48.48	29.03	41.18	25.58	
Higher education	18.18	38.71	23.53	58.14	
**Marital status, %**					0.812 ^b^
Single/Widowed/Divorced	24.24	32.26	23.53	23.26	
Married/Cohabiting	75.78	67.74	76.47	76.74	
**Residential status, %**					0.32 ^b^
Village	36.36	22.58	41.18	41.86	
Town/City	63.64	77.42	58.82	58.14	
**Employment, %**					0.0003 *^b^
Employed	42.42	18.75	35.29	67.44	
Unemployed	0.00	0.00	5.88	0.00	
Pensioner	57.58	81.25	58.58	32.56	

**Notes**: IQR, interquartile range; BMI, Body Mass Index; * *p* < 0.05; ^a^ Kruskal–Wallis test; ^b^ chi-square test.

**Table 2:** Treatment characteristics of the patient cohort.

Variable	Leukemia	Myeloma	Lymphoma	*p*-Value
***n***	33	32	34	
**Duration of treatment,** months, median (IQR)	3.00 (6.00)	8.00 (30.4)	5.5 (17.00)	0.19 ^a^
**Treatment regimen, %**				<0.0001 *^b^
Venetoclax in combination with an anti-CD20 monoclonal antibody (obinutuzumab or rituximab)	63.64	-	-	
Venetoclax monotherapy	6.06	-	-	
Observation	30.30	-	-	
Daratumumab (anti-CD38 monoclonal antibody)	-	18.76	-	
Daratumumab plus bortezomib (anti-CD38 monoclonal antibody combined with a proteasome inhibitor)	-	40.63	-	
Daratumumab plus lenalidomide	-	18.75	-	
Lenalidomide	-	12.50	-	
Teclistamab	-	3.12	-	
Carfilzomib	-	3.12	-	
Elotuzumab	-	3.12	-	
O-CHOP (obinutuzumab, cyclophosphamide, doxorubicin, vincristine, and prednisone)	-	-	41.18	
R-CHOP (rituximab, cyclophosphamide, doxorubicin, vincristine, and prednisone)	-	-	58.82	
**ECOG performance status, %**				0.08 ^b^
0	42.42	15.62	14.70	
1	51.51	71.87	79.41	
2	6.06	9.37	5.88	
3	-	3.12	-	
4	-	-	-	
5	-	-	-	
**Comorbidities, %**				0.25 ^b^
No comorbidities	48.49	43.75	29.41	
Hypertension	33.33	43.75	52.94	
Hypertension and diabetes mellitus	9.09	9.37	17.65	
Hypothyroidism	6.06	-	-	
Immunodeficiency	3.03	-	-	
Rheumatoid arthritis	-	3.13	-	
**Fatigue Severity Scale** median (IQR)	32.00 (30.00)	37.00 (21.00)	30.50 (25.00)	0.35 ^a^

**Notes**: IQR, interquartile range; * *p* < 0.05; ^a^ Kruskal–Wallis test; ^b^ chi-square test.

**Table 3:** Physical fitness according to clinical diagnosis.

Variable	Leukemia (*n* = 33)	Myeloma (*n* = 32)	Lymphoma (*n* = 34)	Control Group (*n* = 43)
2MST	89.00 (22.00)	83.00 (30.00)	82.50 (35)	95.00 (32.00)
30CST	10.00 (4.00)	9.00 (4.00)	11.00 (3.00)	13.00 (6.00)
TUG	8.10 (3.13)	9.45 (5.76)	8.45 (2.58)	6.84 (3.06)

**Notes**: Values are presented as median (IQR). 2MST, Two-Minute Step Test; 30CST, 30-Second Chair Stand Test; TUG, Timed Up and Go Test; IQR, interquartile range.

**Table 4:** Effect of clinical diagnosis on physical fitness in the study groups.

Variable	Kruskal–Wallis Test, *p*-Value	Group 1 vs. Group 4, *p*-Value	Group 2 vs. Group 4, *p*-Value	Group 3 vs. Group 4, *p*-Value	η^2^
2MST	0.0042	NS	0.018 *	0.010 *	0.075
30CST	<0.0001 *	0.002 *	<0.0001 *	0.018 *	0.159
TUG	<0.0001 *	0.032 *	<0.0001 *	0.015 *	0.161

**Notes**: Group 1, leukemia; Group 2, myeloma; Group 3, lymphoma; Group 4, control group. 2MST, 2-Minute Step Test; 30CST, 30-Second Chair Stand Test; TUG, Timed Up and Go test; η^2^, eta squared; * *p* < 0.05.

**Table 5:** Predictors of physical fitness—all subjects.

Model: Dependent Variable, Overall Physical Fitness Score					
Variable	β Coefficient	SE	t	*p*-Value	95% CI
Leukemia vs. control group	−0.485	0.159	−3.05	0.003 *	[−0.799; −0.171]
Myeloma vs. control group	−0.794	0.159	−4.98	<0.001 *	[−1.109; −0.478]
Lymphoma vs. control group	−0.572	0.153	−3.74	<0.001 *	[−0.875; −0.269]
Age, years	−0.028	0.004	−7.08	<0.001 *	[−0.035; −0.020]
Sex: male = 0, female = 1	0.198	0.112	1.77	0.078	[−0.023; 0.419]
BMI, kg/m^2^	−0.022	0.012	−1.85	0.067	[−0.046; 0.002]
**Modelfit**: R^2^ = 0.452, adjusted R^2^ = 0.427; *F* = 18.53; *p* < 0.001 *					

**Notes**: β, regression coefficient; SE, standard error; CI, confidence interval; BMI, body mass index; * *p* < 0.05.

**Table 6:** Predictors of physical fitness in the patient cohort.

Model: Dependent Variable, Overall Physical Fitness Score					
Variable	β Coefficient	SE HC3	t	*p*-Value	95% CI
Myeloma vs. Leukemia	−0.318	0.186	−1.709	0.091	[−0.688; 0.051]
Lymphoma vs. Leukemia	−0.119	0.149	−0.801	0.425	[−0.417; 0.177]
Age, years	−0.027	0.005	−4.723	<0.001 *	[−0.039; −0.016]
Sex: male = 0, female = 1	0.119	0.149	0.799	0.426	[−0.177; 0.416]
BMI, kg/m^2^	−0.003	0.017	−0.192	0.848	[−0.038; 0.031]
Education	0.087	0.080	1.084	0.281	[−0.072; 0.247]
Log_2_ (duration of treatment)	0.005	0.042	0.121	0.904	[−0.079; 0.089]
FSS score	−0.004	0.004	−0.931	0.355	[−0.012; 0.004]
Comorbidities	−0.081	0.087	−0.934	0.353	[−0.254; 0.091]
**Modelfit**: R^2^ = 0.393, adjusted R^2^ = 0.331; *F* = 6.33; *p* < 0.001 *					

**Notes**: β, regression coefficient; SE, Heteroskedasticity-Consistent Standard Errors; CI, confidence interval; BMI, body mass index; * *p* < 0.05.

## Data Availability

The data are available upon reasonable request from the corresponding author.
